# COVID-19 Vaccination: Concerns About Its Accessibility, Affordability, and Acceptability

**DOI:** 10.3389/fmed.2021.647294

**Published:** 2021-07-16

**Authors:** Inayat Ali, Shahbaz Ali, Sehar Iqbal

**Affiliations:** ^1^Department of Social and Cultural Anthropology, University of Vienna, Vienna, Austria; ^2^Independent Researcher, Islamabad, Pakistan; ^3^Department of Environmental Health, Centre for Public Health, Medical University of Vienna, Vienna, Austria; ^4^Department of Nutrition and Dietetics, National University of Medical Sciences, Rawalpindi, Pakistan

**Keywords:** COVID-19, vaccination, immunization, disparities, vaccine hesitancy, refusals, low-income countries, high-income countries

## Abstract

By the mid of June 2021, after an almost 1.5-year-long COVID-19 pandemic that has significantly affected the world in multiple ways, various vaccines against COVID-19 have arrived and started worldwide. Yet, economic, (geo)political, and socio-cultural factors may influence its uptake at individual and country levels. Several issues will (and already have been reported in media) revolve around this vaccination regarding its accessibility, affordability, and acceptability at an individual level and a country level. Given that in this commentary, we provoke a discussion: Who—a country as well as the individuals—would have access to it, and who would economically afford it, and who would accept it? Centering these intriguing questions, we revisit the body of literature that explicates vaccine hesitancy, refusal, and resistance, and we also draw on the current literature and media reports about vaccination against COVID-19. We suggest that these backdrops need essential attention so that everyone can afford, accept, and have access to it. Otherwise, the current risk in the face of a year-old pandemic will continue.

## Introduction

Since the coronavirus disease (COVID-19) has overwhelmed the entire world, the longing and production of a vaccine against it have also significantly increased over time (1). By December 2020, ~200 candidate vaccines for COVID-19 went under preclinical and clinical evaluation (2). After several successful trials, various vaccines, e.g., Pfizer–BioNTech, Moderna, and Oxford–AstraZeneca, have arrived and been started in some countries, such as the United Kingdom and Russia. The European Union (EU) has also commenced the vaccination first vaccinating older people and healthcare providers.

This phenomenal discovery would undoubtedly end the almost 1.5 year-old pandemic. Nonetheless, concerns can be raised about its affordability, accessibility, and acceptability at individual and country levels. Unsurprisingly, economic, (geo)political, and socio-cultural factors may influence the vaccine's uptake: Which country can afford the vaccine and which individuals can have access to it and then will accept/refuse the vaccine?

First, low-income countries, such as Papua New Guinea (PNG) and Pakistan, at the first point cannot afford the COVID-19 vaccine. Second, the vaccine may face critical challenges that may affect its uptake. The underlying reasons include various (a) forms of mistrust—i.e., between citizens and government, between laypeople and global stakeholders, and between governments and global stakeholders; (b) forms of institutionalized inequalities and inequities appropriated at local, national, and global levels; and types of rumors and conspiracy theories about vaccination (1, 3). The routine vaccination uptake in Pakistan is still way behind as per the World Health Organization (WHO) recommended 95% (3). Consequently, viruses like measles and polio are still prevalent in the country and cause severe outbreaks. These complex processes may affect the vaccine's affordability, accessibility, and acceptability, especially in the face of the “infodemic” surrounding COVID-19 (4, 5). For instance, many people in Pakistan have considered the pandemic “Western” “plot,” “political game,” or “fake” (6–8), and consequently, they have refused polio vaccination (9). Therefore, this commentary first provides an overview of the vaccination, the situational analysis of the COVID-19 vaccination, and factors that may affect its uptake, its such as various forms of disparities.

## Vaccination: A Brief Overview

Vaccination emerged as the most effective public health intervention to prevent communicable diseases, save lives, and reduce disease burden. Following the “Pasteur's Germ Theory,” Edward Jenner produced the smallpox vaccine in 1798 (10). Globally, many clinical and public health professionals have been studying the nature of immunological memory for the last 100 years (11). From the public health perspective, vaccination is considered a substantial measure to immunize people for reducing vaccine-preventable diseases (VPDs) (12). The success of vaccination programs highly relies on herd immunity at a population level (13), as an increase in herd immunity may result in a lower intensity of infection in the population and thus a lower risk of infection among unvaccinated persons (14).

Despite the remarkable success of vaccination programs, vaccines are neither 100% efficacious nor 100% effective (15). One common argument frequently found in anti-vaccination literature is that people still get the disease after a vaccine (16). That means the lack of vaccine efficacy or lack of adequate protection are generally used for vaccination failure (17), while a failure indicates that the vaccine has not been administered appropriately for any reason (15). Such incomplete coverage, vaccine–vaccine interactions, and manufacturing-related issues are also crucial factors involved in vaccine failure.

In addition to vaccine failure, “vaccine hesitancy” and vaccine reluctancy are growing in public due to a lack of confidence in the vaccine and those who administer it (3). “Vaccine hesitancy” can be defined as a set of beliefs, attitudes, and behaviors that many people hold to decline, delay, or doubt a vaccine (18). Vaccination came under public suspicion when a large population refused pertussis vaccination in the 1980s, and afterward a decrease in measles, mumps, and rubella (MMR) vaccines (19). Rumors and conspiracy theories have since long been associated with vaccinations across the world, which ultimately affect the vaccination uptake (1, 3, 20–22). These narratives are social phenomena that are meaningful within a context (1, 3). Since this hesitancy affects routine vaccine coverage, ultimately resulting in vaccine-preventable disease outbreaks and epidemics, the success of the current COVID-19 vaccine seems unachievable without dealing with these perceptions and practices.

## COVID-19 Vaccination: Situational Analysis

The 2020 COVID-19 pandemic compelled the global scientific community to find the solution in terms of therapeutics and vaccines to control it. Many vaccine candidates joined the endeavor to produce an effective vaccine (2). Several trials were conducted. And finally, a few candidates were successful in producing and supplying vaccines as vaccination started in several countries, notably in the EU at the end of December 2020.

Concerning the acceptance and vaccination strategies to run COVID-19 immunization programs, numerous surveys, and studies have been conducted. For instance, one study in China was carried out in March 2020 for evaluating the risk perception, impacts of COVID-19, and attitudes, acceptance, and preferences of COVID-19 vaccines. The results showed that around 91.3% adult population reported that they would accept COVID-19 vaccination (23). In contrast, a national cross-sectional survey in the UK found that around 64% of participants are likely to accept the stated vaccine (24). Similarly, a global survey of 19 countries concluded that 71.5% of participants showed a positive response regarding the COVID-19 vaccine (25).

Nonetheless, many people are reluctant to receive the COVID-19 vaccine as they are concerned about side effects. The media reported a recent survey in Germany that only 33% of the population showed a willingness to receive the vaccine; however, they were slightly more hesitant about the side effects (26). While about 19% of people said, they do not want to receive this vaccine at all (26).

Similarly in Pakistan, there are a considerable number of people who are hesitant to get the shot of the COVID-19 vaccine. While revising this paper in May 2021, extremely critical rumors have started, such as those who receive the COVID-19 vaccine will die after 2 years. And, for instance, during our data collection for the project on COVID-19 led by Inayat Ali, one respondent shared, “Today got the second dose of COVID-19. During my visit to the vaccination center, I observed either people are well-aware or full of fear. Everyone was asking which vaccine you are injecting Sinopharm or Astra Zeneca. This double mind standard is set by the media, I guess.”

In this regard, the scientific literature is scant in Pakistan. Yet, two studies have focused on healthcare workers to observe their perceptions of the COVID-19 vaccine. One study focusing on healthcare workers found that out of 5,237 responses, 70.25% accepted COVID-19 vaccination, 24.51% were reluctant thus wanted to delay until more data was available, and 0.05% rejected being vaccinated. Vaccine acceptance was more in young (76%) and female gender (63.3%) who were engaged in a tertiary care hospital (51.2%) and provision of direct patient care (61.3%) (27). The reason women refused to receive the vaccine was due to doubts about the effectiveness of the vaccine (31.48%), while the reason men refused to receive the vaccine was because of previous exposure to COVID-19 (42.19%) and the side effect profile of the vaccine (33.17%). The study obtained responses from 555 doctors 89% of them worked in healthcare facilities where they could encounter COVID-19 patients, and 32% tested positive previously; 81% of them shown acceptance as they wanted to be vaccinated while 19% were not convinced to be vaccinated (27). It was interesting to know that 46% of them were anxious to be offered Astra-Zeneca or Pfizer (28).

### Determinants of Vaccine Hesitancy: Focusing on COVID-19 Vaccination

Several rumors and conspiracy theories have already surfaced about COVID-19 and affected its people's perceptions and preventive behaviors (4, 6, 8). Analogously, old narratives have also emerged concerning the COVID-19 vaccine, such as, about its potentially harmful side effects, illnesses, and even death. An extensive antivaccine content has frequently been shared on social media, which directly or indirectly would shape vaccination opinions and may cause vaccine hesitancy (29).

Individual freedom is a critical factor (30), as it sparks controversy among parents who feel deprived of their freedom to make decisions about their children's health (31). This is a complex decision that involves emotional, socio-cultural, spiritual, and political factors as much as cognitive factors (32). During the current pandemic, about 34% of the British population opposed the government's decision to make vaccination legally compulsory for all people (33).

**Figure 1:**
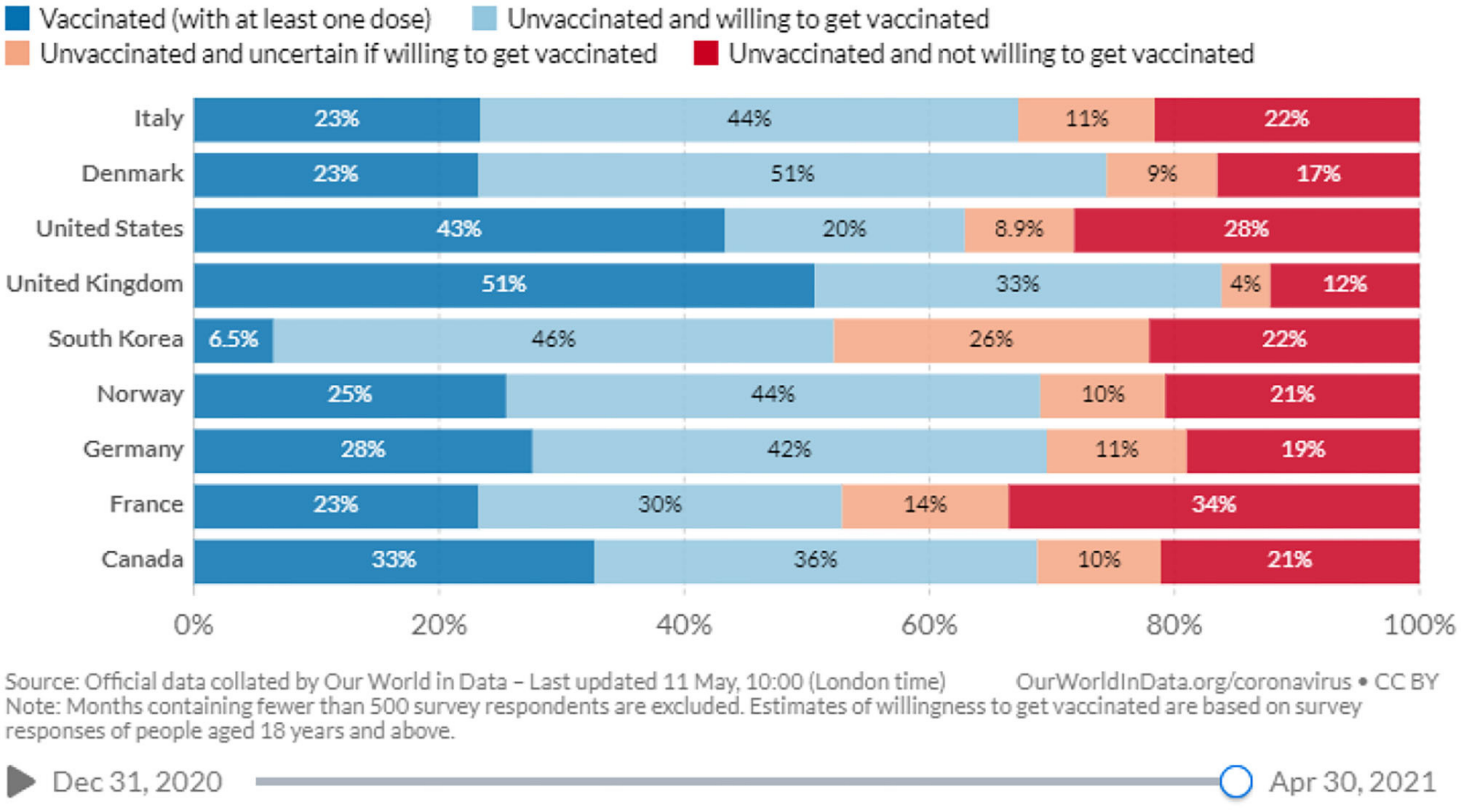
Willingness to get vaccinated against COVID-19 (39).

Trust deficiency is another crucial issue in contemporary society, especially regarding science and knowledge. Many studies of vaccination decision-making and perceptions are closely linked to trust and mistrust in health professionals, government, or public health institutions (3, 6, 34, 35). One study in Italy highlighted that people's trust in science and vaccination decreased between the first and the second phase of the pandemic (36). Moreover, many people refuse to vaccinate in the US due to vaccine-specific concerns, such as the need for more information, anti-vaccine attitudes or beliefs, and a lack of trust (37). Similarly, numerous people in America and Canada preferred natural COVID-19 immunity. They showed mistrust in vaccine benefits and concerns about unforeseen future effects, and commercial profiteering from pharmaceutical companies [(38); see Figure 1].

Adequate knowledge and awareness appear to be another important factor. One recent review reported that lack of knowledge and understanding of the benefits of vaccination, inconsistent recommendations by providers, and uncertainties about cost benefits might be some critical causes of low adult vaccination coverage (40). Similarly, another study found that education about herd immunity and local vaccination coverage could be a useful tool for increasing willingness to vaccinate, generating benefits both to individuals and communities (41).

Likewise, one study from Italy proposed that educational initiatives and other interventions are equally important steps to develop the appropriate awareness in people about COVID-19 vaccination (42).

### COVID-19 Vaccine Affordability: Structured Disparities at Play

By sharing the antibody testing data, one study suggested that about 90% of people are susceptible to COVID-19 and around 60–70% (5.6 billion) of the global population have to be immunized to achieve herd immunity (43). Given that, we reiterate here that will COVID-19 vaccine be available to all people worldwide anxious to accept and want this vaccine? Most probably, it would not be possible due to the existing economic and political conditions of the world.

**Figure 2:**
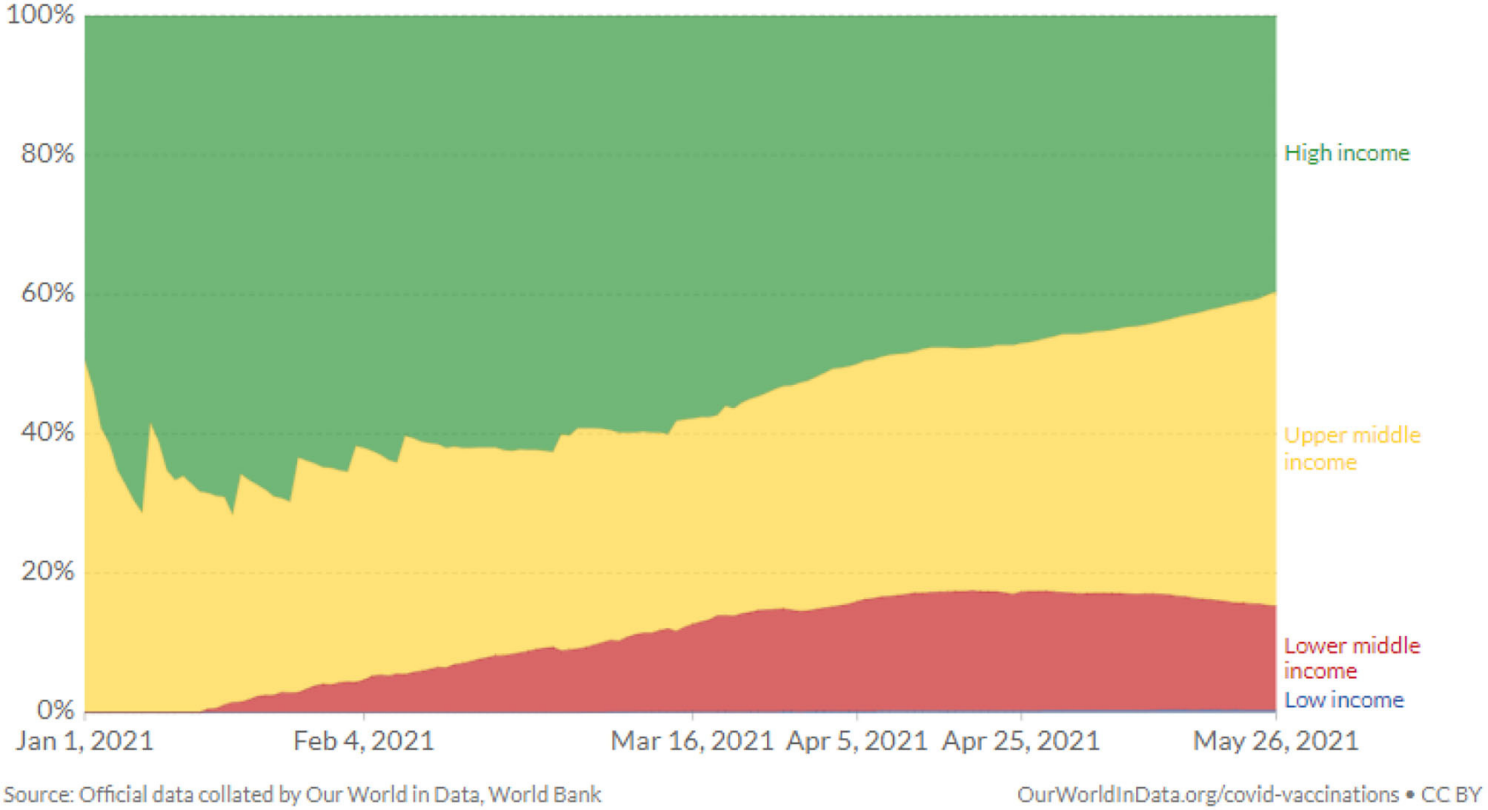
COVID-19 vaccine doses administered by country income group (39).

Recent reports specified that the EU stockpiled around 2 billion vaccine doses, and some high-income countries have already secured large numbers of doses of different candidate vaccines without knowing which one may prove helpful (44). However, low-income countries are far away from the state of purchasing and implementing COVID-19 vaccination programs (see Figure 2). Along with the challenges in resources and manufacturing, there are issues associated with distribution and acceptance. For instance, the vaccine is available in high-income countries as they can afford it, but people refuse to receive it. Here they have a choice to decide either to opt for it or leave it. In contrast, until low-income countries are given vaccines by high-income countries as “donations,” they cannot afford it due to limited economic resources and high population pressure. A small village of Ice Land may have access to an effective vaccine, but the Akha village of Nepal indeed would face significant challenges to have it. At least, this village would not have easy access.

Therefore, the implementation of COVID-19 immunization programs will likely be more affected in low-resource settings. Similarly, it is essential to pursue comprehensive vaccination strategies in parallel to minimize reluctance, hesitancy, and refusal at global, regional, and country levels.

Given these backdrops, it is necessary to reimagine “the culture of public health” (45) since diverse local, national, and global contexts where various economic, (geo)political, and socio-cultural factors are entangled (1). Dealing with these contexts is essential so that every country and individual may quickly and indiscriminately afford, accept, and have access to the COVID-19 vaccine to end over a year-old pandemic. Vaccination proves useful when over 95% of the population receives the vaccine that helps to create what can be called “communal immunity.” Otherwise, even in the presence of an effective COVID-19 vaccine, everyone would be at a similar risk prevalent during these days of the pandemic. The risk multiplies due to globalization since travel is more comfortable than in the past—how a microorganism swiftly travels, we have seen it in the case of COVID-19.

## Conclusion

Based on the Pasture's work, many vaccines have been developed over time. Vaccination done right is a great preventive measure against various communicable diseases, including COVID-19. By revising this paper (in the mid of June 2021), the vaccine against COVID-19, such as Pfizer–BioNTech, Moderna, and AstraZeneca, Sinopharm has arrived and started across the world. However, by writing this paper (December 2020), this vaccination only started in several high-income countries. Like other vaccines, there are genuine concerns about the COVID-19 vaccine who can afford it, who will have access to it, and who will accept it. We provoke that the current vaccination would significantly be determined by the pre-existing socio-cultural, economic, and (geo-)political disparities. Economically rich countries and individuals will have easy access to it, but low-income countries and populations will face significant issues to buy and receive it. We, therefore, suggest that it is important to take these different contexts into account for indiscriminately accessibility, affordability, and acceptability. Otherwise, the same risk that has been visible during the pandemic would continue even in the presence of an effective COVID-19 vaccine.

Moreover, based on the current narratives surrounding the COVID-19 vaccination, we invite ethnographically and sociologically rich accounts to study, analyze, and report the results of factors that not only discourage vaccination uptake but also pay significant attention to those factors that encourage vaccination uptake. Also, there are a plethora of narratives about different vaccines of COVID-19 that which one is more effective. Such detailed studies hold great importance, as these will play a pivotal role to address concerns that shape particular perceptions and practices of vaccination.

## Data Availability Statement

Publicly available datasets were analyzed in this study. This data can be found here: all data are cited.

## Author Contributions

IA: conceptualization, writing the first draft, analysis, revision, and validation. SA: conceptualization, revision, and validation. SI: conceptualization, writing the first draft, and validation. All authors contributed to the article and approved the submitted version.

## Conflict of Interest

The authors declare that the research was conducted in the absence of any commercial or financial relationships that could be construed as a potential conflict of interest.

## References

[1] Ali I. Impact of COVID-19 on vaccination programs: adverse or positive? Hum Vaccin Immunother. (2020) 16:2594–600. 10.1080/21645515.2020.1787065 32961081 PMC7733893

[2] World Health Organization (WHO). DRAFT Landscape of COVID-19 Candidate Vaccines. World Health Organization (2020).

[3] Ali I. Constructing and Negotiating Measles: The Case of Sindh Province of Pakistan. Vienna: University of Vienna (2020).

[4] Ali I. COVID-19: are we ready for the second wave? Disaster Med Public Health Prep. (2020) 14:e16–8. 10.1017/dmp.2020.149 32379015 PMC7239772

[5] Ali I. Anthropology in emergencies: the roles of anthropologists during the COVID-19 pandemic. Pract Anthropol. (2020) 42:16–22. 10.17730/0888-4552.42.4.20

[6] Ali I. Impacts of rumors and conspiracy theories surrounding COVID-19 on preparedness programs. Disaster Med Public Health Prep. (2020) 1–6. 10.1017/dmp.2020.325 32900413 PMC7596562

[7] Ali I Ali S. Why may COVID-19 overwhelm low-income countries like Pakistan? Disaster Med Public Health Prep. (2020). 10.1017/dmp.2020.329. [Epub ahead of print]. 32907694 PMC7674821

[8] Ali I. The COVID-19 pandemic: making sense of rumor and fear. Med Anthropol. (2020) 39:376–9. 10.1080/01459740.2020.1745481 32212931

[9] Ali I Sadique S Ali S. COVID-19 and vaccination campaigns as western plots in Pakistan: government policies, (geo-) politics, local perceptions, and beliefs. Front Sociol. (2021) 6:608979. 10.3389/fsoc.2021.608979 33969047 PMC8102740

[10] Bazin H. A brief history of the prevention of infectious diseases by immunisations. Comp Immunol Microbiol Infect Dis. (2003) 26:293–308. 10.1016/S0147-9571(03)00016-X 12818618

[11] Zinkernagel RM. On natural and artificial vaccinations. Ann Rev Immunol. (2003) 21:515–46. 10.1146/annurev.immunol.21.120601.141045 12500980

[12] Jimenez J. Vaccines—a wonderful tool for equity in health. Vaccine. (2001) 19:2201–5. 10.1016/S0264-410X(00)00447-3 11257333

[13] May T. Public communication, risk perception, and the viability of preventive vaccination against communicable diseases. Bioethics. (2005) 19:407–21. 10.1111/j.1467-8519.2005.00452.x 16222856

[14] Smith PG. Concepts of herd protection and immunity. Proc Vaccinol. (2010) 2:134–9. 10.1016/j.provac.2010.07.005

[15] Heininger U Bachtiar N Bahri P Dana A Dodoo A Gidudu J. The concept of vaccination failure. Vaccine. (2012) 30:1265–8. 10.1016/j.vaccine.2011.12.048 22197579

[16] WorldHealthOrganization (WHO). Six Common Misconceptions About Immunization. Geneva: World Health Organization (WHO) (2013).

[17] Cherry JD. Pertussis: challenges today and for the future. PLoS Pathog. (2013) 9:e1003418. 10.1371/journal.ppat.1003418 23935481 PMC3723573

[18] Peretti-Watel P Larson HJ Ward JK Schulz WS Verger P. Vaccine hesitancy: clarifying a theoretical framework for an ambiguous notion. PLoS Curr. (2015) 7. 10.1371/currents.outbreaks.6844c80ff9f5b273f34c91f71b7fc289 PMC4353679 25789201

[19] Vernon JG. Immunisation policy: from compliance to concordance? Br J Gen Pract. (2003) 53:399–404. Available online at: https://bjgp.org/content/bjgp/53/490/399.full.pdf 12830570 PMC1314602

[20] Feldman-Savelsberg P Ndonko FT Schmidt-Ehry B. Sterilizing vaccines or the politics of the womb: retrospective study of a rumor in cameroon. Med Anthropol Q. (2000) 14:159–79. 10.1525/maq.2000.14.2.159 10879368

[21] Pop CA. Locating purity within corruption rumors: narratives of HPV vaccination refusal in a peri-urban community of southern Romania. Med Anthropol Q. (2016) 30:563–81. 10.1111/maq.12290 26990219

[22] Nichter M. Vaccinations in the Third World: a consideration of community demand. Soc Sci Med. (1995) 41:617–32. 10.1016/0277-9536(95)00034-5 7502096

[23] Wang J Jing R Lai X Zhang H Lyu Y Knoll MD. Acceptance of COVID-19 Vaccination during the COVID-19 Pandemic in China. Vaccines. (2020) 8:482. 10.3390/vaccines8030482 32867224 PMC7565574

[24] Sherman SM Smith LE Sim J Amlôt R Cutts M Dasch H. COVID-19 vaccination intention in the UK: results from the COVID-19 Vaccination Acceptability Study (CoVAccS), a nationally representative cross-sectional survey. Hum Vaccin Immunother. (2021) 17:1612–21. 10.1101/2020.08.13.20174045 33242386 PMC8115754

[25] Lazarus JV Ratzan SC Palayew A Gostin LO Larson HJ Rabin K. A global survey of potential acceptance of a COVID-19 vaccine. Nat Med. (2020) 27:225–8. 10.1038/s41591-020-1124-9 33082575 PMC7573523

[26] Deutsche Welle (DW). Coronavirus: Two-Thirds of Germans Willing to Receive COVID Vaccine. Bonn: Deutsche Welle (DW) (2020).

[27] Malik A Malik J Ishaq U. Acceptance of COVID-19 vaccine in Pakistan among health care workers. medRxiv [Preprint]. (2021). 10.1101/2021.02.23.21252271 PMC8443053 34525110

[28] Gallup Pakistan and the Pakistan Islamic Medical Association (PIMA). National Survey of Potential Acceptance of COVID-19 Vaccine in Healthcare Workers. Islamabad: Gallup Pakistan and the Pakistan Islamic Medical Association (PIMA) (2021). Available online at: https://gallup.com.pk/wp/wp-content/uploads/2021/03/PIMA-Gallup-PR-revised-1.pdf (accessed May 31, 2021).

[29] Puri N Coomes EA Haghbayan H Gunaratne K. Social media and vaccine hesitancy: new updates for the era of COVID-19 and globalized infectious diseases. Hum Vaccin Immunother. (2020) 16:2586–93. 10.1080/21645515.2020.1780846 32693678 PMC7733887

[30] Asveld L. Mass-vaccination programmes and the value of respect for autonomy. Bioethics. (2008) 22:245–57. 10.1111/j.1467-8519.2008.00630.x 18447860

[31] The LID. The imperative of vaccination. Lancet Infect Dis. (2017) 17:1099. 10.1016/S1473-3099(17)30590-X 29115253

[32] Dubé E Laberge C Guay M Bramadat P Roy R Bettinger JA. Vaccine hesitancy: an overview. Hum Vaccin Immunother. (2013) 9:1763–73. 10.4161/hv.24657 23584253 PMC3906279

[33] YouGov. Once a Coronavirus Vaccine is Available, Would You Support or Oppose the Government Making It Legally Compulsory for all People in Britain to Be Vaccinated? UK. (2020). Available online at: https://yougov.co.uk/topics/health/survey-results/daily/2020/11/17/6aa7f/1 (accessed December 28, 2020).

[34] Benin AL Wisler-Scher DJ Colson E Shapiro ED Holmboe ES. Qualitative analysis of mothers' decision-making about vaccines for infants: the importance of trust. Pediatrics. (2006) 117:1532–41. 10.1542/peds.2005-1728 16651306

[35] Brownlie J Howson A. ‘Leaps of faith'and MMR: an empirical study of trust. Sociology. (2005) 39:221–39. 10.1177/0038038505050536

[36] Palamenghi L Barello S Boccia S Graffigna G. Mistrust in biomedical research and vaccine hesitancy: the forefront challenge in the battle against COVID-19 in Italy. Eur J Epidemiol. (2020) 35:785–8. 10.1007/s10654-020-00675-8 32808095 PMC7431109

[37] Fisher KA Bloomstone SJ Walder J Crawford S Fouayzi H Mazor KM. Attitudes toward a potential SARS-CoV-2 vaccine: a survey of US adults. Ann Intern Med. (2020) 173:964–73. 10.7326/M20-3569 32886525 PMC7505019

[38] Taylor S Landry CA Paluszek MM Groenewoud R Rachor GS Asmundson GJ. A proactive approach for managing COVID-19: the importance of understanding the motivational roots of vaccination hesitancy for SARS-CoV2. Front Psychol. (2020) 11:2890. 10.3389/fpsyg.2020.575950 33192883 PMC7604422

[39] Ritchie H Ortiz-Ospina E Beltekian D Mathieu E Hasell J Macdonald B. Coronavirus (COVID-19) Vaccinations. London: Our World in Data (2021). Available online at: https://ourworldindata.org/covid-vaccinations (accessed May 28, 2021).

[40] de Gomensoro E Del Giudice G Doherty TM. Challenges in adult vaccination. Ann Med. (2018) 50:181–92. 10.1080/07853890.2017.1417632 29338438

[41] Logan J Nederhoff D Koch B Griffith B Wolfson J Awan FA. ‘What have you HEARD about the HERD?' Does education about local influenza vaccination coverage and herd immunity affect willingness to vaccinate? Vaccine. (2018) 36:4118–25. 10.1016/j.vaccine.2018.05.037 29789242 PMC6008254

[42] Barello S Nania T Dellafiore F Graffigna G Caruso R. ‘Vaccine hesitancy'among university students in Italy during the COVID-19 pandemic. Eur J Epidemiol. (2020) 35:781–3. 10.1007/s10654-020-00670-z 32761440 PMC7409616

[43] Bloom BR Nowak GJ Orenstein W. When will we have a vaccine? —Understanding questions and answers about covid-19 vaccination. N Engl J Med. (2020) 383:2202–4. 10.1056/NEJMp2025331 32897660

[44] Jeyanathan M Afkhami S Smaill F Miller MS Lichty BD Xing Z. Immunological considerations for COVID-19 vaccine strategies. Nat Rev Immunol. (2020) 20:615–32. 10.1038/s41577-020-00434-6 32887954 PMC7472682

[45] Harrison EA Wu JW. Vaccine confidence in the time of COVID-19. Eur J Epidemiol. (2020) 35:325–30. 10.1007/s10654-020-00634-3 32318915 PMC7174145

