# Corrigendum: COVID-19 Vaccination: Concerns About Its Accessibility, Affordability, and Acceptability

**DOI:** 10.3389/fmed.2021.749023

**Published:** 2021-09-07

**Authors:** Inayat Ali, Shahbaz Ali, Sehar Iqbal

**Affiliations:** ^1^Department of Social and Cultural Anthropology, University of Vienna, Vienna, Austria; ^2^Independent Researcher, Islamabad, Pakistan; ^3^Department of Environmental Health, Centre for Public Health, Medical University of Vienna, Vienna, Austria; ^4^Department of Nutrition and Dietetics, National University of Medical Sciences, Rawalpindi, Pakistan

**Keywords:** COVID-19, vaccination, immunization, disparities, vaccine hesitancy, refusals, low-income countries, high-income countries

In the published article, there was an error regarding the affiliations for Sehar Iqbal. As well as having affiliation 4, they should also have ^3^ Department of Environmental Health, Centre for Public Health, Medical University of Vienna, Vienna, Austria.

The authors apologize for this error and state that this does not change the scientific conclusions of the article in any way. The original article has been updated.

## Publisher's Note

All claims expressed in this article are solely those of the authors and do not necessarily represent those of their affiliated organizations, or those of the publisher, the editors and the reviewers. Any product that may be evaluated in this article, or claim that may be made by its manufacturer, is not guaranteed or endorsed by the publisher.

